# What do we know about treating breast-cancer-related lymphedema? Review of the current knowledge about therapeutic options

**DOI:** 10.1007/s12282-022-01428-z

**Published:** 2022-12-26

**Authors:** Karolina Anuszkiewicz, Jerzy Jankau, Martyna Kur

**Affiliations:** Department of Plastic Surgery, Faculty of Medicine, Medical University of Gdańsk, 80-214 Gdańsk, Poland

**Keywords:** Lymphedema, Breast cancer, Treatment, Prevention

## Abstract

Breast-cancer-related lymphedema (BCRL) is a common consequence of oncological treatment. Its management is a complicated, chronic, and arduous process. Therapeutic options can be divided on non-surgical and surgical methods, although there is still no clear consensus about their effectiveness in preventing or stopping the disease. That brings problems in everyday practice, as there are no guidelines about proper time for starting therapy and no agreement about which management will be beneficial for each patient. The aim of this review is to summarize current knowledge about possible treatment choices, non-surgical so as surgical, indicate knowledge gaps, and try to direct pathways for future studies.

## Introduction

Breast-cancer-related lymphedema (BCRL) is a common consequence of oncological management with incidence after mastectomy procedure about 3–10%. The major risk factors include procedures on the axillary lymph nodes. If sentinel lymph node biopsy (SNB) is applied, the incidence is about 6–8% and increase even to 13–50% if axillary lymph node dissection (ALND) was applied [1–3]. Prevalence in radiotherapy of the axilla area is about 10–15% [2, 4]. International Society of Lymphology classification described four stages of the disease [5]. Stage 0 is a preclinical stage, with no symptoms present, but changes in the lymph flow can be found. Stage I is a visible swelling of the limb, sometimes with pitting edema, and patient may report heaviness or numbness of the arm. Elevation of the affected extremity usually solves or relieves symptoms, which differentiates stage I from stage II, as in stage II elevation does not ameliorate the edema. In stage III, the chronic stage, skin changes appear with hyperplasia, fibrosis, thickening, lichenification, and secondary ulcers [6]. Edema and skin thickening cause problems with the limb mobility, pain and lead to the worsening of the patients' life quality [7]. Lymphedema management is a complicated, chronic, and arduous process. Maladjusted or delayed interventions may lead to further lymphedema progression. Lack of consensus about which patients would benefit from each treatment choice and no guidelines about the proper time for starting therapy cause therapeutic problems. The aim of this review is to summarize current knowledge about possible treatment choices, non-surgical so as surgical, indicate knowledge gaps, and try to direct pathways for future studies.

## Non-surgical therapies

### Physical activity

To this day, several trials investigated the safety of exercises after breast cancer surgical treatment, pointing no negative effect on lymphedema [8, 9]. Box et al. was the first study that showed reduced lymphedema incidence in the exercise group vs. control group (11 vs. 30% in 2 years follow-up) [10]. Their results were partially confirmed by Schmitz et al. as they resulted in 11% incidence of lymphedema in the group training weight lifting compared to 16% in the control group with no physical activity, although no statistical significance was achieved [11]. Additionally, Ding et al. evaluated 26 studies providing favorable outcome in reducing the incidence of lymphedema during physiotherapy, exercise programs, and delayed exercise [12].

As it comes to the treatment of BCRL, in the early stages of the disease, resistance exercises (RE) appeared to have a positive influence on symptoms such as pain or numbness of the limb, muscle strength, and eventually, increase the quality of patients’ life [12, 13]. Meta-analysis performed by Hasenoehrl et al. showed that applying RE can potentially decrease lymphedema, although authors described several limitations, as why those results should be treated with a caution [14]. Trials varied with study protocols, the intensity of proposed training program, methods of evaluating lymphedema, or follow-up time. Different physical activities such as an aqua therapy [15], yoga [16], aerobic exercise [17], pilates [18], and stretching [19] were also evaluated with proven safety but with no influence in prevention or treatment of lymphedema.

Based on the current literature, exercises involving the upper limb progressed slowly appear to be safe for individuals after operative breast cancer treatment and can potentially be effective in the BCRL prevention. In addition, physical activity may bring benefits in the lymphedema management for patients with early stage of the disease as it alleviates the symptoms [20]. Nonetheless, there is still a lack of consensus about the proper time for starting physical activity after surgery, the intensity of a proposed training program, and its frequency.

### Compression therapy

Compression therapy is widely used in lymphedema treatment. The principal rule is gradually decreasing the pressure gradient from the highest in the distal wrist area to the lowest toward the arm, which supposes to facilitate the movement of lymphatic fluid upwards. Commonly used garments have a pressure range of about 15–20 mmHg. No additional benefits were noted with pressure higher than 30 mmHg [21–23]. Compression therapy seems to be efficient in the volume reduction and progression of the early stage lymphedema [24]. Longhurst et al. reported even 80% of patients after breast cancer surgical treatment had been prescribed with compression garments, but instructions for their usage are incoherent, as some patients were advised to wear them only during activities, others during walking hours, some others for a day and a night [25]. Results of multicenter randomized-controlled trial reported in 2021 showed significant improvement in arm lymphedema volume from the addition of a nighttime compression to daytime compression therapy [26]. Necessity of wearing garments during exercises is unclear. Several studies showed that wearing compression while exercising may have no additional positive influence on lymphedema reduction compared to exercises solely, although no adverse effects were also observed [27, 28]. Even 20% of patients discontinue using garments despite doctor’s advice as a consequence of feeling uncomfortable with heat and sweating, reduction of limb’s mobility or no clinical effect [25]. Personally adjusted seamless garments appear to support a better quality of life, although their costs need to be discussed and accepted by the patient [29]. One of the alternatives is Kinesio taping with similar efficiency and being perceived as more comfortable for the patients, although there is a greater risk for skin complications, probably due to self-removal of the tapes [30]. Additional compression technique is intermittent pneumatic compression (IPC). IPC is a device with a pneumatic cuff applied to the limb and connected to the pump. So far, studies suggest that IPC adds no benefits when combined with other treatment options for lymphedema [29, 31, 32].

Compression therapy may be beneficial for individuals with an early stage of the disease [20]. Still, there are no proofs for the effectiveness of wearing compression garments routinely after the surgery as a prevention technique. The type of compression technique should be adjusted to the patient. Continuing compression therapy during the night may be more beneficial than daytime therapy solely. Future studies should conclude if there is necessity of routinely worn garments during physical activity and as a prevention technique if no symptoms of lymphedema are observed.

### Manual lymphatic drainage (MLD)

MLD is a form of physiotherapy in which therapists put a gentle pressure with slow and rhythmical movements on the patient’s body. Areas unaffected with lymphedema are treated first, making it possible for the fluid to move from the decongest regions [33]. Nine systematic reviews with four meta-analyses tried to conclude the MLD effect on lymphedema with inconsistent results [33–41]. Detailed data about each study are presented in Table 1. Shao et al. claimed that adding MLD to the standard therapy could enhance the effectiveness of treating volume reduction, which was partially confirmed by Ezzo et al. However, four performed meta-analysis declined to find such a conclusion, stating MLD cannot significantly reduce or delay BCRL. Partial improvement was seen in patients under 60 years old, with mild lymphedema (stage I), patients who had more than 20 sessions, and if therapy was continued over 1 month. Recently developed method assumed adding indocyanine green (ICG) imaging during MLD to visualize alternative lymphatic pathways. Due to this, MLD is more personally adjusted. So far, there is no trials compering effectiveness of this approach, but it may potentially bring more benefits in BCRL management [42, 43].

**Table 1:** Systematic reviews summarizing trials about MLD

References	Trials included (*N*)	Number of patients (*N*)	Meta-analysis performed?	Results	Conclusion
Muller et al. [34]	8 (5 with BCRL): Beltmonte (2012), Dayes (2013), Gradalski (2015), Odebiyi (2014), Ridner (2013)	236	No	One study reported increased HRQoL among patients randomized to the MLD group. No studies reported reductions in HRQoL, or severe adverse events after MLD	The effect of MLD on the HRQoL of patients with chronic edema is unclear
Huang et al. [37]	10: Andersen (2000), Didem (2005), Johansson (1998), Johansson (1999), McNeely (2004), Sitzia (2002), Williams (2002), Szolnoky (2009), Devoogdt (2011), Torres Lacomba (2010)	566	Yes; 8 studies included	Two studies evaluating the preventive outcome of MLD found no significant difference in the incidence of lymphedema between the MLD and standard treatment groups, with a risk ratio of 0.63 and a 95% confidence interval (CI) of 0.14–2.82. Seven studies assessed the reduction in arm volume and found no significant difference between the MLD and standard treatment groups, with a weighted mean difference of 75.12 (95% CI − 9.34 to 159.58)	The current evidence from RCTs does not support the use of MLD in preventing or treating lymphedema
Ezzo et al. [33]	6: Johansson (1998), Johannson (1999), Andersen (2000), McNeely (2004), Sitzia (2002), Williams (2002)	NM	No	Result I: group I: MLD + physiotherapy vs. group II: physiotherapy solely; improvements in both groups, no difference for percent reduction between groups Result II: group I: MLD + compression bandaging vs. group II: compression bandaging; additional 7.11% reduction for MLD (*p* = 0.06)	MLD is safe and may offer additional benefit to compression bandaging for swelling reduction. Individuals with mild-to-moderate BCRL may be the ones who benefit from adding MLD. In trials where MLD and sleeve were compared with a nonMLD treatment and sleeve, volumetric outcomes were inconsistent within the same trial
Shao et al. [38]	4: Andersen (2000), Dayes (2013), McNeely (2004), Didem (2005)	234	No	There was a significant difference in volume reduction between MLD plus routine treatment and sole routine treatment	Adding MLD to the ST could enhance the effectiveness of treating volume reduction of lymphedema, but might not improve subjective symptoms or arm function
Liang et al. [39]	17: Tambour (2018), Devoogdt (2018), Zhang (2016), Cho (2016), Bergmann (2014), Ridner (2013), Zimmernann (2012), Belmonte (2012), Devoogdt (2011), Szolnoky (2009), Didem (2005), McNeely (2004), Williams (2002), Sitzia (2002), Andersen (2000), Johansson (1999), Johansson (1998)	1911	Yes; 12 studies included	MLD did not significantly reduce lymphedema compared with the control group (standardized mean difference (SMD): 0.09, 95% confidence interval (CI): [0.85 to 0.67]) MLD could significantly reduce lymphedema in patients under the age of 60 years (SMD: 1.77, 95% CI: [2.23 to 1.31]) and an intervention time of 1 month (SMD: 1.77, 95% CI [2.23–1.30]). Meanwhile, 4 RCTs including, 1364 patients, revealed that MLD could not significantly prevent the risk of lymphedema (risk ratio (RR): 0.61, 95% CI [0.29–1.26]) for patients having breast cancer surgery	Meta-analysis of 12 RCTs showed that MLD cannot significantly reduce or prevent lymphedema in patients after breast cancer surgery. However, well-designed RCTs with a larger sample size are required, especially in patients under the age of 60 years or an intervention time of 1 month
Wanchai et al., [36]	10: Andersen (2000), Gradalski (2015), Gurdal (2012), Maher (2012), McNeely (2004), Sitzia (2002), Szolnoky (2009), Williams (2002), Devoogdt (2011), Zimmermann (2012)	NM	No	Based on the results, it cannot be concluded that MLD helps to reduce the risk of BCRL for women after breast surgery. Regarding the effect of MLD on managing BCRL, the findings indicate that MLD alone or MLD combined with other treatments was likely to give similar benefits in terms of reducing arm volume for women diagnosed with BCRL	Scientific evidence to support the benefits of MLD on preventing or reducing BCRL remains unclear. More rigorous studies to confirm findings on the effectiveness of MLD are needed
Thompson et al., [35]	17: Andersen (2000), Belmonte (2012), Bergmann (2014), Gradalski (2015), Ha (2017), McNeely (2004), Johansson (1998), Odebiyi (2014), Ridner (2013), Sanal-Toprak (2019), Sitzia (2002), Tambour (2018), Williams (2002), Cho (2016), Devoogdt (2011), Devoogdt (2018), Zimmermann (2012)	869	No	Some studies reported positive effects of MLD on volume reduction, quality of life, and symptom-related outcomes compared with other treatments, while other studies reported no additional benefit of MLD as a component of complex decongestive therapy. In patients at-risk, MLD was reported to reduce incidence of lymphedema in some studies, while others reported no such benefits	The reviewed articles reported conflicting findings and were often limited by methodological issues. This review highlights the need for further experimental studies on the effectiveness of MLD in lymphedema

*HRQoL* health-related quality of life, *MDL* manual lymphatic drainage, *NM* not mentioned, *BCRL* breast-cancer-related lymphedema, *ST* standard therapy, *RCT* randomized-controlled trials

Based on the current literature, MLD should not be routinely proposed to the patient with BCRL, as its effectiveness seems to be poor. Larger trials with more rigorous study protocols are needed for making a definitive conclusion about this treatment option.

### Complete decongestive therapy (CDT)

CDT is a two-phase program used in the BCRL treatment. Phase I is an “Intensive phase” with a combination of exercises, MLD, bandaging, and skin and nails routine. The sessions take place 5 times a week and last about 2–4 weeks. When a maximal benefit is achieved, the phase II starts as a “Maintenance phase”. It consists of a life-long self-care therapy including compression therapy, exercises, and self-made MLD provided with various frequency [44]. Intensive phase of CDT was proved to have a positive influence on lymphedema reduction with effects seen despite previous treatment strategy [45, 46]. Keskin et al. showed that the benefits were greater if the CDT was applied in the early lymphedema stage (stage I) [45]. Findings were confirmed by Borman et al., who reported that improvements in volume were related negatively with the stage of BCRL, and additionally, with the duration of the disease [47]. Therapeutic effect seems to be increased if the duration of lymphedema is less than 1 year [48]. Several studies investigated the role of MLD in CDT and resulted in a similar improvement of both groups, despite of applying MLD to CDT protocol [46, 49, 50]. The main issue of CDT therapy is its cost and inconvenience for the patient as it requires a lot of time, self-discipline, and strict cooperation with a physiotherapist. As outcomes of “Intensive phase” are good, the “Maintenance phase” is problematic and symptoms of lymphedema may start to get worse over time. Regular group sessions combined with an educational program and healthcare provider supervision seems to be beneficial for sustaining achieved results [51, 52].

Studies confirmed positive role of CDT in the management of BCRL, although the role of MLD in CDT seems to be poor. Future studies should investigate a proper time for starting CDT, as a current literature suggests patients with early stage of the disease and its duration of less than 1 year achieve greater benefits. Protocols for “Maintenance phase” should be designed; thus, the patient could sustain with results achieved.

### Laser therapy

Clinical studies suggest low-level laser therapy (LLLT), described as photobiomodulation (PBM), may reduce inflammation and prevent fibrosis. LLLT acts mainly on cytochrome c oxidase in the mitochondria, facilitates electron transport, and increases adenosine triphosphate (ATP) production. In animal model, LLLT decreased COX-2 expression and inflammatory infiltrate. Antioxidant balance is inherent in the reduction of fibrosis; moreover, LLLT may stimulate lymphangiogenesis [53]. Number of trials reported favorable effect of LLLT in volume and pain reduction so as shoulder mobility in patients with BCRL compared to IPC, MLD, Sham laser or no treatment [54, 55]. Though, when compared to other types of active interventions, LLLT did not improve outcomes significantly [56]. Further, no long-term results of LLLT therapy were announced. Achieving general conclusion is tough, as studies vary with the protocols. Different parameters of LLLT were used with dosage from 0.3 J/cm^2^ to 2.4 J/cm^2^ and with a time from 20 s per one point to 1 min. Treated area was most commonly axillary region, but also cubital fossa and in some publications authors described it as “limb region” with no specific details [57]. In many studies, standard of reporting laser parameters was poor and not coherent with World Association of Laser Therapy (WALT) recommendations. Positive findings include no side effects of laser therapy and potential time saving compared to other treatment options. In in vitro model, LLLT did not affect the modification of human breast adenocarcinoma cells, including their clonogenic efficiency [58].

LLLT could be potentially effective and time-saving option in BCRL treatment, although there is a clear need for larger, well-designed randomize-controlled trials with detailed protocol and parameters reported according to WALT recommendations. Long-term follow-up of patients treated with LLLT is indicated to conclude if the therapeutic effect is sustained over time.

### Pharmacology

The role of pharmacology in lymphedema management is based on immunological processes, which contribute to this condition and it can be potentially useful in both—the prevention and the treatment. Lymphedema is a chronic inflammatory state, which leads to fibrosis. Therapies could potentially influence on two pathways: prevention of a fibrosis or promoting lymphangiogenesis. Skin and lymph nodes from the region affected of lymphedema appear to have increased levels of macrophage, dendric cells, and particularly, CD4 + cells [59, 60]. Studies reported that animal models with no CD4 + cells did not develop lymphedema after lymph node removal [61, 62]. Gardenier et al. showed that topical therapy with tacrolimus in a mouse model prevented secondary lymphedema and decreased edema in a group with late treatment onset after disruption of lymph vessels in a tail [63]. Inhibiting Th2 cell differentiation with monoclonal antibodies of interleukin-4 (IL) and IL-13 improved lymphatic function and also decreased fibrosis [64]. Transforming growth factor-beta1 (TGF-beta1) was found to be another regulator of fibrosis after lymph vessels damage and could be potential target for novel therapies [65]. Studies on small animals provided another potential pathway of treating lymphedema by promoting lymphangiogenesis with the usage of vascular endothelial growth factor D and C (VEGF-D; VEGF-C). VEGF-C seems to have superior lymphangiogenic activity and less side effects such as seromas [66]. Its activity was described by Visuri et. al, who used VEGF-C and VEGF-C156S on pig models after detriment of inguinal lymph vessels. Both factors induced lymphangiogenesis with no signs of angiogenesis [67]. In 2020, Phase I of study on Lymfactin^®^ was announced. Lymfactin^®^ is an adenovirus type-5-based gene therapy involving expression of human VEGF-C in the damaged tissue. The drug was administrated in fifteen patients with BCRL; 12-months follow-up showed good tolerance of Lymfactin^®^. The study is continued and follow-up after 36 months and 5 years is planned [68].

Target therapies are promising and potentially effective option in BCRL management. Their advantage is acting on the pathology of the edema, not symptoms like remaining non-surgical treatment options. Further studies should develop those findings, confirm safety of potential drugs and their benefits.

## Surgical methods

### Axillary reverse mapping (ARM)

Positive SNB is a standard indication for ALND which is an independent risk factor for BCRL. Axillary reverse mapping (ARM) is a technique which supposes to decrease this risk. ARM is based on the assumption that drainage from the breast and the arm is carried out by the different lymphatic pathways and contains different axillary lymph nodes [69]. Using the markers injected subcutaneously in the arm area (for example, ICG, blue dye, radioisotope) can indicate which lymph nodes and lymphatic channels should be kept to minimalize the possibility of lymphedema incidence. Meta-analysis made by Guo et al. concluded reduction of the BCRL in the patients treated with ALND combined with ARM. However, the number of patients included was small (37 persons) [70]. In 2019, Beek et al. reported outcomes of randomized-controlled trial of ARM in patients with diagnosed breast cancer with clinically negative lymph nodes (CN-) although with positive SNB. Patients were divided into ALND group (*n* = 46) and ALND with preserving ARM lymph nodes (*n* = 48). ARM-ALND reported fewer symptoms related to BCRL in 2-years follow-up [71]. The major concern is keeping the oncological clearance as connections between breast and arm lymphatic pathways exist. In positive SNB with CN-, ARM nodes were metastatic in 0–11% of patients [72]. In patients with CN +, those percentages increase even up to 60% [72]. Beek et al. reported that neoadjuvant chemotherapy decreased the number of ARM metastatic lymph nodes to 16.5% [73]. However, those numbers are still high and do not allow for preserving ARM lymph nodes in CN + patients.

ARM technique has promising results as a prevention method. However, oncological safety should be determined. Future studies should indicate in which patient ARM procedure can be safely proposed and if preserving ARM lymph nodes requires additional approach in the treatment and supervising the patient. Techniques helping to estimate involvement of ARM should be developed.

### Lymphovenous anastomosis (LVA)

LVA is a microsurgical technique based on the connection between lymphatic vessels and collateral veins, which allows bypassing obstructed lymph channels. As studies suggest LVA is beneficial in both, preserving and treating BCRL [74, 75]. Circumference of the affected limb improved in 65–100% of the patients after LVA procedure with a median volume reduction of 67%. Improvement was also seen in the quality of life and subjective feelings of the symptoms [75, 76]. Moreover, prophylactic LVA performed immediately after ALND appeared to decrease BCRL incidence from 30 to 3% with results sustained in 18 months follow-up [77]. Currently, a study called LYMPHA is recruiting patients for ALND and ALND with same-time LVA for making a conclusion about effectiveness of LVA as a prophylactic technique [78]. A potential problem in this approach is the influence of theoretical postoperative radiotherapy or chemotherapy on anastomosis efficiency. Proper patients’ selection is crucial for a positive outcome. Poumellec et al. found a greater reduction in volume and circumference measurements in patients with stage II vs. stage III, with no improvement in patients with stage IV [79]. Findings were confirmed by Chang et al.[80]. To perform LVA, lymphatic vessels need to be free from fibrosis; thus, the procedure is indicated for the patient with mild stages of the disease. Surgical techniques vary from end-to-end, side-to-end, or end-to-side anastomosis. So far, only one study compared those techniques, with more favorable effect for end-to-side vs. end-to-end anastomosis [81]. The number of anastomoses in the studies varies from 1.6 to 9 but usually counts 3 or more [74, 82]. There is no proven correlation between number of anastomoses and clinical outcomes. It appears that quality and diameter are more important for the effect, but no study has proven this theory yet. As yet there are no standards for postoperative care. Usage of bandages or garments varies from 4 weeks to 6 months after the surgery [83]. Data about the possible discontinuation of non-surgical therapies after surgery are incomplete, and the percentage of patients who were able to desist from non-invasive methods ranges from 30 to 100% [81, 84].

Given the present literature findings, LVA is efficient in treating mild stages of BCRL and has promising outcomes in preventing edema. Longer follow-up is needed to determine the long-term efficiency of the anastomosis with possible thrombosis or sclerosis complications. Data about postoperative management are incomplete and require future studies with particular emphasis on the need of non-surgical methods after the surgery.

### Vascularized lymph node transfer (VLNT)

VLNT is a procedure dedicated for the patients, who are in lack of functional lymph nodes in the affected limb and with lymphatic vessels no longer proper for LVA due to sclerosis. Current literature indicates that VLNT is efficient and gives BCRL volume reduction by 40% in about 90% of the patients. Additionally, procedure has positive influence on patients' life quality and the number of skin infections [85, 86]. Mechanism of action is still unknown, yet there are two major hypotheses. Transferred lymph nodes act like a “sponge”, adsorb lymphatic fluid, and redirect it into the vascular system. Second theory is based on production of VEGF-C by the transferred tissue, which induces lymphangiogenesis [87]. Lymph nodes are transplanted with the flap vascularized by the donor vessels. The number of transplanted lymph nodes depends on the donor area and individual anatomical variability, and it usually is about 3 in the groin area to even up to 7 in omental area [88]. Recipient region can be orthotropic (axilla) or heterotopic (elbow, wrist). Orthotropic placement can improve scar release with its replacement with well-vascularized tissue and can be performed together with post-mastectomy breast reconstruction [89]. Nonetheless, this area is often affected by prior operations/radiotherapy; thus, the anatomy may be changed, tissue may be fibrotic, and preparation of the recipient vessels may be more difficult. Moreover, if the lymphedema is in the distal limb area, the drainage may be insufficient. In those cases, heterotopic placement may be more efficient as it is facile with the gravity, although esthetic outcome is usually poorer with more visible scars [88]. However, meta-analysis made by Chocron et al. found non-inferiority between axilla and wrist as a recipient site in a limb circumference [90]. Choosing the donor site may bring difficulties as there are lack of studies comparing outcomes for each region. Potential problems and advantages for described harvest areas are presented in Table 2 [91, 92].So far, choosing the donor area is mainly based on patient's medical history with potential contraindications for specific region and surgeon experience and preference. Need of compression therapy after the procedure is still unclear. About 40% of patients were able to resign of garments completely and another 56% could limit compression therapy session to one per week [85, 93].

**Table 2:** Characteristic of flaps used in VLNT

Donor region	Lymph nodes group	Donor vessels	Advantages	Disadvantages
Groin	Superficial inguinal lymph nodes	Superficial circumflex iliac artery and vain	Possible same-time breast reconstruction; avoidance of additional scar for lymph node harvest Scar hidden by clothing Large skin paddle can be included	Possible iatrogenic lymphedema in the lower limb–evaluation of lymph circulation is required before the procedure Risk of donor-site seroma
Submental	Cervical lymph nodes level Ia/Ib	Submental artery	Low risk for donor-site lymphedema Large number of lymph nodes Small scar hidden in the wrinkles	Variability of anatomy, risk of mandibular nerve injury Risk of submandibular gland injury Partial resection of platysma muscle
Supraclavicular	Cervical lymph nodes level Vb	Branch of transvers cervical vessels; branches of the external jugular vein	Low risk for donor-site lymphedema	Injury of supraclavicular nerve (potential paresthesia) Risk of thoracic duct injury Risk of phrenic nerve injury Risk of Horner syndrome More visible scar
Omental	Omental lymph nodes	Gastroepiploic artery and vein	Low risk for donor-site lymphedema Possible laparoscopic harvest—small scar Large number of lymph nodes	No skin paddle (no possible monitoring of the flap recovery) Possible abdominal complications: hernias, peritonitis, damage of intra-abdominal organs, bowel perforation, internal bleeding Visceral vessels-flaccid pedicle prone to twisting
Mesenteric	Mesenteric lymph nodes	Branches of superior mesenteric artery	Low risk for donor-site lymphedema Possible laparoscopic harvest—small scar Large number of lymph nodes	No skin paddle (no possible monitoring of the flap recovery) Possible abdominal complications: hernias, peritonitis, damage of intra-abdominal organs, bowel perforation, internal bleeding, bowel obstruction Visceral vessels-flaccid pedicle prone to twisting

### Lipectomy

Fibroadipose soft tissue, which develops in stage IV lymphedema, can be removed in debulking procedures. If the fibrotic changes of the skin are large, excisional approach called Charles procedure is sometimes required; however, it leads to large scars and requires skin transplant. Potential complication may lead to recurrence of the lymphedema, further skin grafts, and poor cosmetic outcome [94]. Suction-assisted lipectomy is a less invasive procedure [89]. First, area is infiltrated with the solid of Ringer's lactate, adrenaline, and lidocaine. Lipoaspiration starts from the distal parts and move upwards circumferentially [95]. The procedure is effective and decreases the limb circumference immediately. However, the effect is not permanent and requires long-life-supporting therapy with compression garments. Lipectomy may be complemented to VLNT, especially for the patient with advanced BCRL stages. Studies showed improvement of arm circumference, if lipectomy was added after the VLNT procedure in patients with stage II and III of the lymphedema [96] (Table 3).

**Table 3:** Proposition of interventions suitable for each stage of the disease

Stage	Intervention
Prophylaxis	Sentinel lymph node biopsy before ALND Considering ARM Mastectomy combined with LVA Same-time breast reconstruction combined with VNLT Exercises after the surgical procedure
Patient with a high risk of BCRL or with stage 0 disease	Exercises Compression garments Consideration of LVA
Patient in stage I/II	Exercises Compression garments CDT LVA/VNLT
Patient in stage III/IV	Lipectomy; additionally, with VNLT

## Discussion

Breast-cancer-related lymphedema is a common complication of oncological treatment, especially if axillary lymph nodes procedures were applied. Although its incidence is high, there are still no guidelines for the medical practitioners, on how to deal with this condition. First, lymphedema is diagnosed based mainly on clinical symptoms. Some of the authors described lymphedema as a greater volume of the limb, others as a larger circumference compared to unaffected extremity. In several studies, authors used lymphangiography to detect abnormal lymphatic flow. The lack of one accepted definition of lymphedema leads to different study protocols and eventually makes outcomes impossible to compare. Patients are usually treated with multiple therapies, or have been treated before, which gives another problem with the conclusion about the effectiveness of one specific therapeutic approach. The acting of non-surgical methods is based on alleviation of the symptoms related to BCRL; thus, they do not heal the cause of the disease and eventually patient requires life-long therapy with strict cooperation and self-discipline. Direct patient-born costs of BCRL (i.e., cost of garments, physiotherapy) were estimated at 2306$ per year. Indirect costs (i.e., work absence, productivity losses) were evaluated for an additional 3325$ annually. Moreover, during a 2-year postoperative period, patients with BCRL required more hospitalization and specialized medical care, and eventually seven-time higher healthcare charge compared to patient without BCRL [97]. This indicates the great need for expanding the methods of preventing and treating BCRL. Development of microsurgery brought promising results in BCRL management, as it was the first method focused on pathophysiologic nature of the condition. Outcomes of the patients were favorable and, importantly, a high number of patients were able to discontinue tedious non-surgical therapies. Prophylactic LVA performed with same-time mastectomy and ALND were estimated to reduce expected lifetime patient cost to 6295$ [98]. The major problem remains with the access to the microsurgery. Microsurgical training is a long and expensive process and requires experienced surgeon [98]. Given into consideration, the high incidence of BCRL, performing microsurgical technique in every patient with this condition is probably nowadays unachievable. For this reason, surgical approach to BCRL, despite its effectiveness, is second-line option. Currently, developing microsurgery skills seems to be an inherent part of surgical specialization. Studies showed that learning microsurgery on the basic level may be achieved by self-learning, using solely tutorial videos as a guide [99]. This creates a great possibility, especially for young doctors, and contribute to the more common availability of microsurgery procedures. We hope that, in the future, microsurgery could be part of a standard protocol for BCRL prevention in women with a high risk of lymphedema. However, microsurgical reconstructions on the lymphatic vessels are not without disadvantages. The major problem remains to be the complications in the harvest area in VNLT. The patients’ follow-ups are too short for judging the long-term patency and effectiveness of LVA. The following issue is proper patient selection, and what is more important, choosing a proper time for the interventions. According to the literature, practically all treatment options were more effective if had been implemented in the early stage of the disease [79, 80]. This indicates a great need for a proper screening and importance of control visits with a detailed physical examination. So far, we lack examination which helps us to predict which patient will develop lymphedema. Moreover, we lack tool which allows us to estimate which patient will remain with stage 0/I of the disease and in which one, the edema will progress. How much time can we wait to conclude that non-invasive methods are not sufficient for this patient. Proper diagnostic approach and patient screening process are further fields in BCRL, which should be developed. This would allow for fast intervention, and hopefully increase quality of life of the patients with BCRL.
